# Pathways to reproductive autonomy: Using path analysis to predict family planning outcomes in the United States

**DOI:** 10.1111/hsc.14094

**Published:** 2022-11-01

**Authors:** Laura E. T. Swan, Shelby E. McDonald, Sarah K. Price

**Affiliations:** ^1^ Department of Population Health Sciences University of Wisconsin‐Madison Madison Wisconsin USA; ^2^ Virginia Commonwealth University Clark‐Hill Institute for Positive Youth Development Richmond Virginia USA; ^3^ Virginia Commonwealth University School of Social Work Richmond Virginia USA

**Keywords:** contraceptive use, healthcare access, reproductive autonomy, unintended pregnancy

## Abstract

In the United States, about half of pregnancies are unintended, and most women of reproductive age are at risk of unintended pregnancy. Research has explored predictors of contraceptive use and unintended pregnancy, but there is a lack of research regarding access to *preferred* contraceptive method(s) and the complex pathways from sociodemographic factors to these family planning outcomes. This study applied Levesque et al.'s (2013) healthcare access framework to investigate pathways from sociodemographic factors and indicators of access to family planning outcomes using secondary data. Data were collected at four time points via an online survey between November 2012 and June 2014. Participants were US women of reproductive age who were seeking to avoid pregnancy (*N* = 1036; M_age_ = 27.91, SD = 5.39; 6.9% Black, 13.6% Hispanic, 70.2% white, 9.4% other race/ethnicity). We conducted mediational path analysis, and results indicated that contraceptive knowledge (*β* = 0.116, *p* = 0.004), insurance coverage (*β* = 0.423, *p* < 0.001), and relational provider engagement (*β* = 0.265, *p* = 0.011) were significant predictors of access to preferred contraceptive method. Access to preferred contraceptive method directly predicted use of more effective contraception (*β* = 0.260, *p* < 0.001) and indirectly predicted decreased likelihood of experiencing unintended pregnancy via contraceptive method(s) effectiveness (*β* = −0.014, 95% confidence interval: −0.041, −0.005). This study identifies pathways to and through access to preferred contraceptive methods that may be important in determining family planning outcomes such as contraceptive use and unintended pregnancy. This information can be used to improve access to contraception, ultimately increasing reproductive autonomy by helping family planning outcomes align with patients' needs and priorities.

**What is known about this topic**

- Unintended pregnancy is common and is associated with negative health and mental health outcomes.
- Preventing unintended pregnancies and promoting reproductive autonomy requires access to contraception.
- Healthcare access is multidimensional and requires approachable, acceptable, available, affordable and appropriate care.

**What this paper adds**

- Contraceptive knowledge, insurance coverage and provider engagement predicted access to the preferred contraceptive method, which emphasises the approachability, affordability and appropriateness dimensions of healthcare access.
- Access to the preferred contraceptive method directly predicted use of more effective contraception; it also mediated the relationships between predictors (contraceptive knowledge, insurance coverage and provider engagement) and family planning outcomes (contraceptive method(s) effectiveness and unintended pregnancy).

## 1 INTRODUCTION

Unintended pregnancy is associated with lower quality of life and with increased distress, depression and anxiety for the pregnant person during and after pregnancy and is also linked to poor maternal and child health outcomes (Gipson et al., 2008; Leathers & Kelley, 2000; Schwarz et al., 2008). In the United States, 40%–45% of pregnancies are unintended (Ahrens et al., 2018; Finer & Zolna, 2016), and a majority of women of reproductive age are at risk of unintended pregnancy (i.e. having sex with a man and desiring to avoid pregnancy; Jones et al., 2015).

Nationally representative data suggest that, over the course of a year, about 85% of heterosexual couples not using contraception will become pregnant (Trussell, 2011). When used consistently and correctly, user‐dependent hormonal contraceptive methods (i.e. vaginal ring or contraceptive pill, patch or injection) can be highly effective, preventing over 99% of pregnancies (Guttmacher Institute, 2015; National Health Service, 2020). However, when these methods are used incorrectly or inconsistently, their effectiveness declines (Guttmacher Institute, 2015; NHS, 2020). US women who use contraception consistently and correctly account for only 5% of unintended pregnancies, whereas inconsistent contraceptive use accounts for 41% of unintended pregnancies (Guttmacher Institute, 2015). Some contraceptive methods, such as natural family planning methods (e.g. monitoring monthly fertility patterns and timing sexual encounters to avoid pregnancy) and withdrawal, are highly susceptible to user error, resulting in variable effectiveness (NHS, 2020; Urrutia et al., 2018). In contrast, sterilisation and long‐acting reversible contraception (LARC), including the contraceptive implant and intrauterine devices (IUDs) are highly effective methods (greater than 99% effective) with greatly reduced opportunities for user failure (Guttmacher Institute, 2015; NHS, 2020).

### 1.1 Guiding framework

Contraceptive access promotes reproductive autonomy and reduces the risk of unintended pregnancy. Levesque et al.'s (2013) healthcare access framework is useful in understanding the many factors influencing contraceptive access. This framework conceptualises access to healthcare as determined by five dimensions that interact to generate healthcare access: (1) approachability, (2) acceptability, (3) availability and accommodation, (4) affordability and (5) appropriateness. Applying this framework to contraceptive access provides a holistic approach to examining the many barriers to and facilitators of access to contraception.

According to Levesque et al.'s (2013) framework, the first dimension of healthcare access is *approachability*, which requires community members to perceive the need for care. In particular, the availability of contraceptive information and the transparency of contraceptive services impact the approachability of contraceptive care (Bessett et al., 2015). Lack of contraceptive knowledge is a common barrier to contraceptive access, as many US women lack information about birth control and misestimate its effectiveness (Cabral et al., 2018; Frost et al., 2012). Women with more contraceptive knowledge are more likely to use contraception and to use more effective methods than women with less contraceptive knowledge (Frost et al., 2012; Guttmacher Institute, 2015; Guzzo & Hayford, 2018).

The second dimension of healthcare access, *acceptability*, refers to the ability to seek care, with health beliefs and social norms either promoting contraceptive access or creating stigma, shame and lack of trust in healthcare systems (Holt et al., 2020; Levesque et al., 2013). Over half of US women of reproductive age consider pregnancy avoidance very important, and almost a quarter would be very unhappy to become pregnant (Jones, 2017). However, 36% of US women of reproductive age report some degree of pregnancy fatalism, indicating that many women believe that the occurrence or timing of pregnancy is outside of their control (Jones, 2018). Research suggests that contraceptive use is predicted by pregnancy fatalism and by the importance of pregnancy avoidance to the contraceptive user, with women who desire to and believe they can prevent pregnancy being more likely to use contraception (Frost et al., 2012; Hamidi et al., 2018; Jones, 2017).

The third dimension of Levesque et al.'s (2013) framework, *availability and accommodation*, refers to the ability to physically reach care in a timely manner. This dimension of contraceptive access is influenced by the availability of over‐the‐counter contraception options, the distance patients must travel for services, clinic hours of operation and the availability of transportation needed to reach care (Holt et al., 2020; Kennedy et al., 2019; Levesque et al., 2013). These issues can produce barriers to contraceptive care (Pratt et al., 2014), especially for low‐income people and those living in rural areas (Beeson et al., 2014; Sundstrom et al., 2019).

The fourth dimension of healthcare access, *affordability*, refers to patients' ability to pay for care, which depends on patient income, access to health insurance and the cost of services (Levesque et al., 2013). The cost of family planning services and lack of health insurance are some of the most common barriers to contraceptive care (Pratt et al., 2014; Zimmerman, 2017). Health insurance is a key aspect of contraceptive affordability (Levesque et al., 2013; Swan et al., 2020) with studies showing that women with insurance are more likely to use contraception and to use more effective contraception than uninsured women (Kavanaugh et al., 2020; Nearns, 2009). A dramatic change to contraceptive affordability has occurred over the past decade, related to the Affordable Care Act (ACA). The ACA expanded health insurance coverage and Medicaid eligibility and mandated contraceptive coverage as a preventive service (Redhead & Kinzer, 2015; Sonfield, 2011). The ACA has led to increased contraceptive affordability by decreasing out‐of‐pocket contraceptive costs (e.g. Finer et al., 2014; Sonfield et al., 2015) and uninsurance rates (e.g. Decker et al., 2018; Johnston & McMorrow, 2020). Furthermore, states that took advantage of optional Medicaid expansion under the ACA have seen increased public insurance coverage (e.g. Boudreaux et al., 2019; Gibbs et al., 2020), decreased uninsurance (e.g. Boudreaux et al., 2019; Hale et al., 2018), and decreased cost as a barrier to care (Johnston et al., 2018).

Finally, *appropriateness* refers to patients' ability to engage in their contraceptive care (Levesque et al., 2013). In order for patients to engage with providers for appropriate contraceptive care, providers must be adequately trained and prepared to provide unbiased care that matches their patients' needs and priorities (Casey & Gomez‐Lobo, 2013; Gomez et al., 2014; Holt et al., 2020; Swan et al., 2020). Scholars and public health advocates have recommended that healthcare providers assess patients' reproductive life plans and tailor contraceptive decision‐making support based on patients' needs and priorities (Casey & Gomez‐Lobo, 2013; Dehlendorf et al., 2014; Holt et al., 2017). Research shows that when patients experience this sort of provider engagement during contraceptive counselling, they are more likely to initiate or continue contraceptive use and maintain the use of a highly effective method (Dehlendorf et al., 2016; Lee et al., 2015).

### 1.2 Study purpose

**FIGURE 1:**
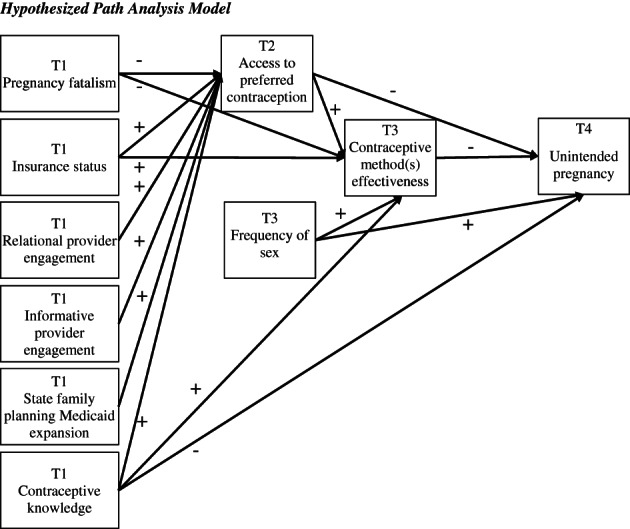
Hypothesised path analysis model. Model adjusted for the effects of age, age^2^, race/ethnicity and education. T1 = Time 1; T2 = Time 2; T3 = Time 3; T4 = Time 4

Most family planning research has traditionally focused on contraceptive uptake, adherence, and/or effectiveness. While these are important family planning outcomes, they are reflective of broader public health goals rather than indicators of reproductive autonomy. In order to understand the multifaceted barriers to reproductive autonomy as well as their relationships with more traditional family planning outcomes, the current study focused on the ability to access *preferred* contraceptive methods and the complex pathways from sociodemographic factors and indicators of access to traditional family planning outcomes (i.e. contraceptive effectiveness and unintended pregnancy). This study applied Levesque et al.'s (2013) healthcare access framework to investigate direct and indirect pathways from pregnancy fatalism, insurance status, provider engagement, family planning Medicaid expansion and contraceptive knowledge to family planning outcomes. Broadly, we hypothesised that these predictors would have a direct effect on contraceptive method(s) effectiveness and unintended pregnancy as well as an indirect effect via the ability to access preferred contraception. The conceptual model and hypothesised effects are shown in Figure 1, and the specific hypotheses are listed in Appendix A.

## 2 MATERIALS AND METHODS

### 2.1 Data and sample

We used panel data collected by the Guttmacher Institute (n.d.) using address‐based sampling. Data were collected at four time points from November 2012 to June 2014. This online survey collected information about contraceptive use, pregnancy motivation and healthcare access. Participants were US women aged 18–39 years who, at baseline, had ever had vaginal sex with a man, were not pregnant, had not had a tubal ligation, and whose main sexual partner had not had a vasectomy. Respondents chose whether to take the survey in English or Spanish and received $10 compensation for each completed wave. We received confirmation from the Virginia Commonwealth University Institutional Review Board (IRB) that this secondary data analysis did not require IRB review due to not qualifying as human subject's research.

There were 6 months between each survey wave, with 4634 women completing wave 1, 3207 women completing wave 2, 2398 women completing wave 3 and 1842 women completing wave 4. Participants who completed all four waves were more likely than those who dropped out of the study to be older, insured, have access to their preferred contraceptive method, have lower education and pregnancy fatalism, and use more effective contraception. Participants identifying their race/ethnicity as white or other were more likely to complete all four survey waves whereas those identifying as Black or Hispanic were more likely to drop out before wave four. See Appendix B for more study attrition information.

Women who were actively trying to get pregnant or reported that pregnancy avoidance was not important to them were excluded from the current study. Removal of these cases from the dataset yielded a sample size of 1247. Only cases with complete data for each variable were included in the current analysis (*N* = 1036). Among those who participated in all four survey waves, there was a low rate of missing data (i.e. 0.2%–1.0%), although 14.7% of participants skipped the item assessing the ability to access preferred contraception. Participants who were older, uninsured, Black, lacking provider engagement, using less effective contraception and having sex less frequently were more likely to be missing data on this variable. See Appendix B for more information about missing data.

### 2.2 Variables

#### 2.2.1 Contraceptive knowledge

Contraceptive knowledge was assessed at wave one by asking how much participants knew about pregnancy prevention methods. Response options ranged on a 6‐point Likert‐type scale from “I know nothing” (=0) to “I know everything” (=5). This variable reflects the approachability dimension of healthcare access.

#### 2.2.2 Pregnancy fatalism

Pregnancy fatalism was measured in wave one by asking how much participants agreed with the statement: “It doesn't matter whether I use birth control, when it is my time to get pregnant, it will happen.” Response options ranged from “strongly disagree” (=0) to “strongly agree” (=4). This variable reflects the acceptability dimension of Levesque et al.'s (2013) healthcare access framework.

#### 2.2.3 Insurance status

Insurance status was assessed in wave one using two items. Participants were asked about their health insurance coverage, and those reporting insurance coverage were asked if they had coverage throughout the past 6 months. A dichotomous variable was created with participants who had insurance for all of the last 6 months coded as insured (=1) and participants reporting no insurance or gaps in insurance coded as uninsured (=0). This variable reflects the affordability dimension of healthcare access.

#### 2.2.4 Family planning medicaid expansion

Using the 2011 Title X Family Planning Annual Report (Fowler et al., 2012), a dichotomous variable was created indicating whether, in November/December 2012, participants lived in a state that had expanded family planning Medicaid in the previous year (=1) or lived in a state without expansion (=0). Along with insurance status, this variable reflects the affordability dimension of healthcare access.

#### 2.2.5 Provider engagement

In wave one of the survey, we measured participants' engagement with healthcare providers with four items, which were split into two composite variables that assessed different aspects of provider engagement: (1) relational provider engagement and (2) informative provider engagement. Participants who did not report visiting a women's health provider in the past 6 months were coded as not experiencing provider engagement (=0) and those reporting visiting such a provider were asked follow‐up questions about provider engagement.

Relational provider engagement reflected encounters in which healthcare providers assessed and satisfactorily met patient needs. This variable was assessed by asking how much participants agreed: (1) that the people working at the facility tried to find out their healthcare needs and (2) that they were satisfied with the care they received there. Participants who reported “strongly agree” and/or “somewhat agree” for either or both items were coded as experiencing relational provider engagement (=1), and those reporting “strongly disagree” and/or “somewhat disagree” for both items were coded as not experiencing relational provider engagement (=0).

Informative provider engagement reflected encounters in which healthcare providers shared contraceptive information with patients. This variable was assessed by asking participants if, at their last women's health visit: (1) their provider spent time talking with them about plans for having/not having children and (2) they got information about birth control/pregnancy prevention. Participants who reported “yes” for either or both items were coded as experiencing informative provider engagement (=1), and those reporting “no” for both items were coded as not experiencing informative provider engagement (=0). These variables capture the appropriateness dimension of healthcare access.

#### 2.2.6 Access to preferred contraceptive method

We used a dichotomous variable assessed at wave two to indicate whether or not participants were able to access their preferred contraceptive method. Participants were asked, “If you did not have to worry about cost and could use any type of contraceptive method available, would you want to use a method/different method?” We coded women responding “no” to this question (I would not switch/use a method) as having access to their preferred contraceptive method (=1) and women responding “yes” (I would switch/use a method) or “unsure” as not having access to their preferred contraceptive method (=0). This variable conceptualisation is consistent with Levesque et al.'s (2013) healthcare access framework, which takes into account the multifaceted aspects of healthcare access, allowing participants to self‐report their hypothetical contraceptive method with barriers to access removed. Women reporting that they would not switch methods have indicated that they are currently using their preferred method (out of those methods in existence and those that they are aware of). This approach is in line with recent calls for research that measures reproductive autonomy by considering the use of preferred contraceptive method(s) rather than only focusing on the use of highly effective methods or successful pregnancy prevention (Bryson et al., 2021; Kavanaugh & Pliskin, 2020).

#### 2.2.7 Frequency of sex

The frequency of sex that could lead to conception was measured with a single item: “In the past 30 days, approximately how many times did you have sex with a man?” Response options were: never (=0), 1 time (=1), 2–5 times (=2), 6–10 times (=3) and 11 or more times (=4). This variable was measured at wave three because sexual behaviour was hypothesised to influence same‐time contraceptive behaviour and future unintended pregnancy.

#### 2.2.8 Contraceptive method(s) effectiveness

Past 30 days contraceptive use was measured at wave three using several items, which were used to create a five‐level ordinal contraceptive method(s) effectiveness variable ranging from 0 to 4, with higher numbers corresponding to consistent use of more effective contraceptive methods. Participants who reported using any of 12 contraceptive methods (pill, patch, ring, injectable, implant, IUD, withdrawal, condoms, spermicide, natural family planning, vasectomy and tubal ligation) were then asked about their method‐specific consistency of use.

Participants with implants, IUDs, tubal ligation or partners with vasectomies were coded as long‐acting or permanent contraceptive users (=4). Participants who reported using the pill, patch, ring or contraceptive shot were coded as user‐dependent hormonal contraceptive users (=3) as long as they reported missing 0–1 pills or starting the method on time or 1 day late. Participants who reported slightly imperfect use of user‐dependent hormonal methods (e.g. 2–4 days of missed pills) but also reported using a rhythm/barrier method during the last 30 days were also classified as consistent user‐dependent hormonal contraceptive users (=3). Participants who used the same barrier method every time they had sex or reported using multiple rhythm/barrier methods in a way that suggested at least one method was used every time they had intercourse were categorised as barrier contraceptive users (=2). Participants who reported inconsistent use of one of the above methods were coded as inconsistent contraceptive users (=1). Finally, participants who reported that they did not use any of the above methods or reported only using withdrawal or natural family planning were coded as non‐users of contraception (=0).

#### 2.2.9 Unintended pregnancy

Unintended pregnancy was assessed using three items at wave four. The first item asked about participants' experiences with pregnancy since the prior survey wave, with response options: “I had a miscarriage,” “I had an abortion,” “I had a baby,” “I'm currently pregnant,” “I might be pregnant,” and “none of the above.” Participants reporting a pregnancy since the previous wave were then asked, “Right before you became pregnant this time, did you yourself want to have a(nother) baby at any time in the future?” Participants responding “yes” to this item were asked about the timing of their pregnancy. If they reported no pregnancy or being unsure if they were pregnant, they were coded as not experiencing an unintended pregnancy (=0). Participants reporting a pregnancy and responding “yes” for wanting a baby were coded as no unintended pregnancy (=0) if they answered, “at about the right time” or “later than you wanted” for the timing of the pregnancy and were coded as experiencing an unintended pregnancy (=1) if they answered, “too soon.” Participants reporting a pregnancy and reporting “no,” “do not know,” or “did not care” for wanting a baby were coded as experiencing an unintended pregnancy (=1).

#### 2.2.10 Demographic variables

Race/ethnicity, education and age were included as control variables due to known disparities in family planning outcomes based on these factors (Finer & Zolna, 2011, 2016). These demographic characteristics were compiled prior to data collection as a part of the information used to target the survey to eligible participants. Race/ethnicity categories included: white (reference group), Black, Hispanic and other. Education was coded as high school or less, some college and bachelor's degree or higher (reference group). Age was a continuous variable with values ranging from 18 to 39. Age squared was also included to more accurately model the potentially nonlinear effect of age (Reid & Allum, 2019).

### 2.3 Data analysis

After describing our study sample, we conducted path analysis in Mplus 8.0 using the robust weighted least squares (WLSMV) estimator to assess the direct and indirect pathways from baseline pregnancy fatalism, insurance status, provider engagement, contraceptive knowledge and family planning Medicaid expansion to access to preferred contraception, contraceptive method(s) effectiveness and unintended pregnancy. Frequency of sex was also included as a predictor of contraceptive method(s) effectiveness and unintended pregnancy. Age, age squared, education and race/ethnicity were included as control variables. Grounded in theoretical and conceptual knowledge about these constructs, some direct pathways from the baseline to outcome variables were constrained,^1^ as shown in Figure 1. For all analyses, we used a preestablished significance level of 0.05.

## 3 RESULTS

### 3.1 Descriptive statistics

As shown in Table 1, the majority of the sample identified their race/ethnicity as white (70.2%, *n* = 727), and the average age was 27.91 (SD = 5.39). Participants had relatively low levels of pregnancy fatalism (*M* = 1.37, SD = 1.41) and high levels of self‐reported contraceptive knowledge (*M* = 3.23, SD = 0.95) and most had health insurance (82.0%, *n* = 652). Participants were more likely to report relational provider engagement (62.9%, *n* = 652) than informative provider engagement (42.0%, *n* = 435). Slightly more participants reported the ability to access their preferred contraceptive method (52.0%, *n* = 539) than reported not being able to access their preferred method (48.0%, *n* = 497). Contraceptive method(s) effectiveness was relatively evenly distributed, although more participants consistently used user‐dependent hormonal methods (45.8%, *n* = 475) than no method (17.0%, *n* = 176), inconsistent method use (7.5%, *n* = 78), barrier method only (12.1%, *n* = 125), or long‐acting/permanent method (17.6%, *n* = 182). Most participants (95.4%, *n* = 988) did not experience unintended pregnancy at the time point in question.

**TABLE 1:** Descriptive statistics for path analysis sample (*N* = 1036)

Variable	*n* (%)	*M* (SD)
Race/ethnicity^a^		
Black	71 (6.9%)	
Hispanic	141 (13.6%)	
White	727 (70.2%)	
Other	97 (9.4%)	
Age (range: 18–39)^a^		27.91 (5.39)
Education^a^		
High school	136 (13.1%)	
Some college	376 (36.3%)	
Bachelor's degree	524 (50.6%)	
Pregnancy fatalism (range: 0–4)^a^		1.37 (1.41)
Insurance status^a^		
Uninsured	186 (18.0%)	
Insured	850 (82.0%)	
Relational provider engagement^a^		
No	384 (37.1%)	
Yes	652 (62.9%)	
Informative provider engagement^a^		
No	601 (58.0%)	
Yes	435 (42.0%)	
Contraceptive knowledge (range: 0–5)^a^		3.23 (0.95)
Family planning Medicaid expansion^a^		
No	275 (26.5%)	
Yes	761 (73.5%)	
Access to preferred contraceptive method^b^		
No	497 (48.0%)	
Yes	539 (52.0%)	
Frequency of sex (range: 0–4)^c^		2.09 (1.25)
Contraceptive method(s) effectiveness^c^		
No effective method used	176 (17.0%)	
Inconsistent use	78 (7.5%)	
Barrier method only	125 (12.1%)	
User‐dependent hormonal method	475 (45.8%)	
Long‐acting/permanent	182 (17.6%)	
Unintended pregnancy^d^		
No	988 (95.4%)	
Yes	48 (4.6%)	

^a^ Time 1.

^b^ Time 2.

^c^ Time 3.

^d^ Time 4.

### 3.2 Path analyses

The hypothesised model had good fit for the data (χ^2^ = 8.049, *df* = 9, *p* = 0.5292, comparative fit index [CFI] = 1.00, Tucker‐Lewis index [TLI] = 1.00, root mean square error of approximation [RMSEA] < 0.000 [90% CI: 0.000, 0.032], weighted root mean square residual [WRMR] = 0.381). As shown in Table 2, the model indicated statistically significant direct effects of insurance status (*β* = 0.423, *p* < 0.001), relational provider engagement (*β* = 0.265, *p* = 0.011), and contraceptive knowledge (*β* = 0.116, *p* = 0.004) on the ability to access the preferred contraceptive method. Pregnancy fatalism had a direct and negative effect on contraceptive method(s) effectiveness (*β* = −0.109, *p* = 0.002). Insurance status (*β* = 0.325, *p* = 0.001), contraceptive knowledge (*β* = 0.098, *p* = 0.004), frequency of sex (*β* = 0.071, *p* = 0.032), and access to preferred contraception (*β* = 0.260, *p* < 0.001) had a direct and positive effect on contraceptive method(s) effectiveness. High school education (*β* = 0.628, *p* = 0.002), contraceptive knowledge (*β* = 0.158, *p* = 0.023), and frequency of sex (*β* = 0.222, *p* = 0.009) had a direct and positive effect on unintended pregnancy, and contraceptive method(s) effectiveness (*β* = −0.461, *p* < 0.001) had a direct and negative effect on unintended pregnancy. These findings are also shown in a figure in Appendix C.

**TABLE 2:** Path analysis direct effects (*N* = 1036)

Dependent variable	←Independent variable	*β*	*SE*	*p*‐value
Contraceptive access	Race/ethnicity (ref = white)			
	Black	−0.241	0.170	0.141
	Hispanic	0.034	0.126	0.779
	Other	−0.068	0.142	0.620
	Age	0.068	0.084	0.403
	Age^2^	−0.001	0.001	0.400
	Education (ref = bachelor's degree)			
	High school	−0.072	0.132	0.569
	Some college	−0.168	0.097	0.073
	Pregnancy fatalism	0.071	0.031	0.088
	Insurance status	0.423	0.113	<0.001***
	Relational provider engagement	0.265	0.108	0.011*
	Informative provider engagement	−0.148	0.106	0.146
	FP Medicaid expansion	0.003	0.094	0.977
	Contraceptive knowledge	0.116	0.044	0.004**
Contraceptive use	Race/ethnicity (ref = white)			
	Black	−0.040	0.150	0.772
	Hispanic	−0.098	0.122	0.387
	Other	−0.069	0.121	0.542
	Age	0.071	0.073	0.298
	Age^2^	−0.001	0.001	0.320
	Education (ref = bachelor's degree)			
	High school	0.158	0.119	0.156
	Some college	0.075	0.086	0.351
	Pregnancy fatalism	−0.109	0.027	0.002**
	Insurance status	0.325	0.108	0.001**
	Contraceptive knowledge	0.098	0.038	0.004**
	Frequency of sex	0.071	0.029	0.032*
	Access to preferred contraception	0.260	0.045	<0.001***
Unintended pregnancy	Race/ethnicity (ref = white)			
	Black	0.449	0.973	0.579
	Hispanic	0.076	0.271	0.736
	Other	0.197	0.922	0.797
	Age	0.165	0.207	0.338
	Age^2^	−0.003	0.004	0.364
	Education (ref = bachelor's degree)			
	High school	0.628	0.242	0.002**
	Some college	0.240	0.203	0.156
	Contraceptive knowledge	0.158	0.088	0.023*
	Frequency of sex	0.222	0.082	0.009**
	Access to preferred contraception	0.106	0.095	0.264
	Contraceptive method(s) effectiveness	−0.461	0.141	<0.001***

* *p* < 0.05.

** *p* < 0.01.

*** *p* < 0.001.

Standardised specific indirect effect estimates (see Table 3) indicated that insurance status (*β* = 0.110, 95% CI: 0.056, 0.201), relational provider engagement (*β* = 0.069, 95% CI: 0.018, 0.143) and contraceptive knowledge (*β* = 0.030, 95% CI: 0.011, 0.064) had an indirect effect on contraceptive method(s) effectiveness via access to preferred contraception. The ability to access the preferred contraceptive method (*β* = −0.120, 95% CI: −0.253, −0.076), frequency of sex (*β* = −0.033, 95% CI: −0.075, −0.003), contraceptive knowledge (*β* = −0.045, 95% CI: −0.121, −0.017), pregnancy fatalism (*β* = 0.050, 95% CI: 0.015, 0.087) and insurance status (*β* = −0.150, 95% CI: −0.384, −0.057) had an indirect effect on unintended pregnancy via contraceptive method(s) effectiveness. Finally, contraceptive knowledge (*β* = −0.045, 95% CI: −0.121, ‐0.017), insurance status (*β* = −0.051, 95% CI: −0.127, −0.026) and relational provider engagement (*β* = −0.032, 95% CI: −0.088, −0.009) had an indirect effect on unintended pregnancy via both access to preferred contraception and contraceptive method(s) effectiveness.

**TABLE 3:** Path analysis specific indirect effects (*N* = 1036)

					CI 95%	
Dependent variable	← Mediator(s)	← Independent variable	*β*	*SE*	Lower limit	Upper limit
Contraceptive use	Access	Pregnancy fatalism	0.019	0.009	−0.002	0.033
	Access	Insurance status	0.110	0.037	0.056*	0.201*
	Access	Relational PE	0.069	0.032	0.018*	0.143*
	Access	Informative PE	−0.039	0.030	−0.102	0.015
	Access	FP Medicaid expansion	0.001	0.026	−0.052	0.052
	Access	Contraceptive knowledge	0.030	0.014	0.011*	0.064*
Unintended pregnancy	Contraceptive use	Access	−0.120	0.045	−0.253*	−0.076*
	Contraceptive use	Frequency of sex	−0.033	0.018	−0.075*	−0.003*
	Access	Contraceptive knowledge	0.012	0.018	−0.011	0.059
	Contraceptive use	Contraceptive knowledge	−0.045	0.026	−0.121*	−0.017*
	Contraceptive use ← Access	Contraceptive knowledge	−0.014	0.009	−0.041*	−0.005*
	Access	Pregnancy fatalism	0.008	0.008	−0.006	0.027
	Contraceptive use	Pregnancy fatalism	0.050	0.019	0.015*	0.087*
	Contraceptive use ← Access	Pregnancy fatalism	−0.009	0.005	−0.020	0.001
	Access	Insurance status	0.045	0.055	−0.042	0.175
	Contraceptive use	Insurance status	−0.150	0.085	−0.384*	−0.057*
	Contraceptive use ← Access	Insurance status	−0.051	0.026	−0.127*	−0.026*
	Access	Relational PE	0.028	0.039	−0.024	0.129
	Contraceptive use ← Access	Relational PE	−0.032	0.020	−0.088*	−0.009*
	Access	Informative PE	−0.016	0.026	−0.084	0.018
	Contraceptive use ← Access	Informative PE	0.018	0.017	−0.008	0.059
	Access	FP Medicaid expansion	0.000	0.017	−0.038	0.034
	Contraceptive use ← Access	FP Medicaid expansion	0.000	0.015	−0.029	0.030

*Note*: Access, ability to access to preferred contraceptive method; Contraceptive use, effectiveness of contraceptive method(s) in use; FP, family planning; PE, provider engagement.

* CI indicates significant specific indirect effect.

## 4 DISCUSSION

The aim of this study was to investigate direct and indirect pathways from sociodemographic factors and predictors of healthcare access to family planning outcomes. Our results indicate that contraceptive knowledge, insurance coverage and relational provider engagement are significant predictors of the ability to access the preferred contraceptive method and that there is an indirect effect of access to preferred contraception on unintended pregnancy. These findings support theoretical suppositions about factors that are key to contraceptive access and extend previous work showing relationships between individual family planning constructs.

Previous research suggests that health insurance (Kavanaugh et al., 2020; Nearns, 2009), provider engagement (Dehlendorf et al., 2016; Lee et al., 2015), and contraceptive knowledge (Frost et al., 2012; Guzzo & Hayford, 2018) are important predictors of family planning outcomes. This study corroborates these findings and extends previous research by suggesting that these are also important predictors of access to preferred contraception and that access to the preferred contraceptive method may be the mechanism by which these factors impact family planning outcomes. In this sample, which had relatively high levels of self‐reported contraceptive knowledge, this knowledge played an important role in determining the ability to access the preferred contraceptive method, use of more effective contraceptive method(s), and unintended pregnancy. Results also indicated the importance of pregnancy fatalism in determining contraceptive use and unintended pregnancy. These findings have important implications, as increasing contraceptive knowledge and addressing fatalistic views of pregnancy have the potential to impact access to and use of contraception.

Future studies could explore how self‐reported contraceptive knowledge, health beliefs and self‐efficacy compare to the accuracy of contraceptive knowledge and how these variables interact to impact family planning outcomes. Future research could also investigate how interventions to decrease pregnancy fatalism and increase contraceptive self‐efficacy might influence contraceptive access and ultimately family planning outcomes. The type and source of contraceptive information also likely play an important role in these outcomes, and future research could examine how these factors influence beliefs about pregnancy and reproduction and ultimately impact reproductive autonomy.

This study also explored the role of healthcare provider engagement in predicting family planning outcomes. Our findings indicated that provider engagement based in relationship played a more important role in determining these outcomes than that focused on the provision of information. This is an important contribution of this study, as provider engagement is often considered a one‐dimensional construct, and many interventions targeting family planning outcomes focus on providing patients with non‐personalised contraceptive information (e.g. Langston et al., 2010; Mwaikambo et al., 2011), sometimes through automated messaging (e.g. Smith et al., 2015) that removes the opportunity for patient‐provider engagement. Although contraceptive information is certainly an important piece of contraceptive access, it may be important for healthcare providers to focus on building patient‐provider relationships and providing quality provider engagement rather than simply providing contraceptive information. Providers can offer more appropriate contraceptive care and build patient‐provider relationship by following recommendations for assessing patients' reproductive life plans and providing contraceptive counselling using a shared decision‐making model (Casey & Gomez‐Lobo, 2013; Dehlendorf et al., 2014; Holt et al., 2017; Lee et al., 2015). Future research is needed to better understand the nuance involved in patient‐provider encounters and the role of the patient‐provider relationship in determining contraceptive access and family planning outcomes. Future research could also focus on developing more rigorous measures of provider engagement and examining the dimensionality of this construct. Such an instrument could be used to measure provider engagement across longer time frames and to explore the importance of receiving care via in‐person versus virtual interactions.

Surprisingly, in this study, family planning Medicaid expansion did not significantly predict access to preferred contraception or indirectly predict contraceptive method(s) effectiveness or unintended pregnancy. This was an unexpected finding considering that cost is a commonly cited barrier to contraceptive care (Pratt et al., 2014; Zimmerman, 2017), and family planning Medicaid expansion should, theoretically, decrease the cost of contraception for low‐income Americans. Further, existing research suggests that expanded family planning Medicaid increases contraceptive use (Dunlop et al., 2016; Sonfield & Gold, 2011) and decreases unintended pregnancy (Adams et al., 2015; Sonfield & Gold, 2011). It is possible that the dichotomous variable measuring expanded family planning Medicaid was unable to capture the complexities of how Medicaid expansion impacts contraceptive access, as factors such as moving across state or county lines, or the type of eligibility expansion enacted, could influence how family planning Medicaid affects contraceptive access and family planning outcomes. Furthermore, the current analysis used a 12‐month time lag, with any state with expanded family planning Medicaid in the previous year coded as expanded. It is possible that this did not allow for full maturation of the expansion program, as Frost et al. (2006) recommend a three‐year time lag for such programs to become fully effective. Future research could assess family planning Medicaid expansion using a greater time lag or could capture program maturation by using an ordinal or continuous family planning Medicaid expansion variable.

This study also indicates that access to preferred contraception impacts contraceptive use and, indirectly, unintended pregnancy. This is an area for continued research as few studies have explicitly investigated access to preferred contraception or included it as a mediating variable. This is particularly important because studies examining only the direct relationship between these variables could draw incorrect conclusions about the mechanisms involved in contraceptive preferences, use and efficacy. This study indicates that the relationship between these variables is complex and may require analyses that allow for indirect pathways or interaction effects. Better understanding these relationships could help justify the need for interventions to improve contraceptive access and test the efficacy of these initiatives.

### 4.1 Strengths and limitations

Several limitations should be considered when interpreting these findings. As with most longitudinal studies, some participants who initially responded to the survey did not participate in all four waves. In this case, only 40% of the baseline sample was retained through all four waves, and participants who dropped out between waves one and four differed on key variables from those who completed all four waves. As described elsewhere (Jones et al., 2015), the study recruitment partners, Growth from Knowledge, estimate that 10% of this loss was due to panel turnover, in which people are dropped from the panel after a certain amount of time. Accounting for this panel turnover, the remaining attrition is more comparable to other national longitudinal studies (e.g. Wright, 2003). Nonetheless, this survey attrition is a notable limitation of this analysis, compromising the representativeness of the data and potentially introducing attrition bias.

Aside from this survey attrition, there was a low level of missing data on study variables. However, one key variable, access to preferred contraception, contained 14.7% missing data and missingness on this variable was predicted by other study variables (see Appendix B). Participants may have been more likely to skip this item because of the sensitivity of the subject matter or the theoretical complexity of the question. This is a noteworthy limitation that impacts the validity of our findings. Future research could explore alternative item wording and measurement techniques to simplify the item and potentially increase its response rate.

Scholarship in this area would also benefit from more precise and validated measurement. Several study variables used single items or unvalidated measures, impacting the validity of the study findings. Additionally, the current analysis used a five‐level ordinal measure of contraceptive use, moving from least to most effective contraceptive method. This strategy is similar to methods used in existing research (Mutua et al., 2019; O'Rourke et al., 2008; Rocca & Harper, 2012; Steinberg et al., 2013). Although the use of ordinal variables can limit analysis options and increase opportunities for measurement error, it also allows for the retention of maximum information in situations where it is impossible to observe or calculate numerical values across an interval or ratio scale; moreover, sufficient analysis methods exist to handle this type of variable (Li, 2014; Nussbeck et al., 2006). Considering the benefits and limitations of using ordinal variables, future research could develop and compare alternative methods for measuring contraceptive use and consolidating this information in ways that capture the maximum amount of information.

Finally, these data were collected from 2012 to 2014. This produces findings related to contraceptive access at a critical moment in time when the family planning landscape was shifting related to the implementation of the Affordable Care Act. Continued policy changes have occurred since this data collection, and additional research is needed to replicate these analyses in more contemporary samples.

In addition to these limitations, this study has several important strengths. We are among the first to leverage path analysis methodology to study complex multivariate pathways to family planning outcomes. This study also draws important connections between common family planning outcomes (contraceptive effectiveness and unintended pregnancy) and more accurate and contemporary reflections of reproductive autonomy (preferred contraceptive use). There are few national data sources available containing these nuanced measures of reproductive autonomy across multiple survey waves, and as a result, there have been few longitudinal studies examining reproductive autonomy.

## 5 CONCLUSION

This study identifies pathways to and through access to preferred contraceptive methods that may be important in determining family planning outcomes such as contraceptive use and unintended pregnancy. In particular, contraceptive knowledge, insurance coverage and relational provider engagement were important predictors of the ability to access the preferred contraceptive method. This information can be used to improve access to contraception, ultimately increasing reproductive autonomy by helping family planning outcomes align with patients' needs and priorities.

## AUTHOR'S CONTRIBUTION

The initial idea for this paper came from LS. SM and SP helped finalise the variable conceptualisation and analysis plan. SM provided methodological consultation, and LS ran the analysis. LS drafted the manuscript with guidance and edits from SM and SP. Final edits and subsequent revisions were led by LS with input from all authors.

## CONFLICT OF INTEREST

The authors have no conflict of interest to declare.

## Supporting information


Appendix S1–S3

## Data Availability

The data that support the findings of this study are from the Guttmacher Institute's Continuity and Change in Contraceptive Use Study. The data can be requested at https://www.guttmacher.org/population‐center/dataset/2012‐2014‐continuity‐and‐change‐contraceptive‐use‐study.

## Endnote

^1^
Comparing this hypothesized model to an alternative, unconstrained model with all direct effects estimated from baseline to outcome variables revealed similar model fit (χ^2^ = 0.591, *df* = 1, *p* = 0.4421, CFI = 1.00, TLI = 1.00, RMSEA = 0.000 [90% CI: 0.000, 0.075], WRMR = 0.108). A chi‐square test for difference testing comparing the fit of these two nested models was statistically non‐significant (χ^2^ = 7.441; *df* = 8; *p* = 0.4898), supporting the constrained hypothesized model.

## References

[hsc14094-bib-0001] Adams, E. K. , Galactionova, K. , & Kenney, G. M. (2015). Medicaid family planning waivers in 3 states: did they reduce unwanted births? INQUIRY, 52, 1–15. 10.1177/0046958015588915 PMC5813652 26044941

[hsc14094-bib-0002] Ahrens, K. A. , Thoma, M. E. , Copen, C. E. , Frederiksen, B. N. , Decker, E. J. , & Moskosky, S. (2018). Unintended pregnancy and interpregnancy interval by maternal age, National Survey of Family Growth. Contraception, 98(1), 52–55. 10.1016/j.contraception.2018.02.013 29501647 PMC6379777

[hsc14094-bib-0003] Beeson, T. , Wood, S. , Bruen, B. , Goldberg, D. G. , Mead, H. , & Rosenbaum, S. (2014). Accessibility of long‐acting reversible contraceptives (LARCs) in Federally Qualified Health Centers (FQHCs). Contraception, 89(2), 91–96. 10.1016/j.contraception.2013.09.014 24210278

[hsc14094-bib-0004] Bessett, D. , Prager, J. , Havard, J. , Murphy, D. J. , Agénor, M. , & Foster, A. M. (2015). Barriers to contraceptive access after health care reform: Experiences of young adults in Massachusetts. Women's Health Issues, 25(2), 91–96. 10.1016/j.whi.2014.11.002 25630846

[hsc14094-bib-0005] Boudreaux, M. , Choi, Y. S. , Xie, L. , & Marthey, D. (2019). Medicaid expansion at Title X clinics: Client volume, payer mix, and contraceptive method type. Medical Care, 57(6), 437–443. 10.1097/MLR.0000000000001120 30973473 PMC6522286

[hsc14094-bib-0006] Bryson, A. , Koyama, A. , & Hassan, A. (2021). Addressing long‐acting reversible contraception access, bias, and coercion: Supporting adolescent and young adult reproductive autonomy. Current Opinion in Pediatrics, 33, 345–353. 10.1097/MOP.0000000000001008 33797464

[hsc14094-bib-0007] Cabral, M. A. , Schroeder, R. , Armstrong, E. M. , El Ayadi, A. M. , Gürel, A. L. , Chang, J. , & Harper, C. C. (2018). Pregnancy intentions, contraceptive knowledge and educational aspirations among community college students. Perspectives on Sexual and Reproductive Health, 50(4), 181–188. 10.1363/psrh.12081 30376215

[hsc14094-bib-0008] Casey, F. , & Gomez‐Lobo, V. (2013). Disparities in contraceptive access and provision. Seminars in Reproductive Medicine, 31(5), 347–359. 10.1055/s-0033-1348893 23934695

[hsc14094-bib-0009] Decker, E. J. , Ahrens, K. A. , Fowler, C. I. , Carter, M. , Gavin, L. , & Moskosky, S. (2018). Trends in health insurance coverage of Title X Family planning program clients, 2005‐2015. Journal of Women's Health, 27(5), 684–690. 10.1089/jwh.2017.6465 PMC6750757 29237143

[hsc14094-bib-0010] Dehlendorf, C. , Henderson, J. T. , Vittinghoff, E. , Grumbach, K. , Levy, K. , Schmittdiel, J. , Lee, J. , Schillinger, D. , & Steinauer, J. (2016). Association of the quality of interpersonal care during family planning counseling with contraceptive use. American Journal of Obstetrics and Gynecology, 215(1), 78.e1–78.e9. 10.1016/j.ajog.2016.01.173 26827879

[hsc14094-bib-0011] Dehlendorf, C. , Krajewski, C. , & Borrero, S. (2014). Contraceptive counseling: Best practices to ensure quality communication and enable effective contraceptive use. Clinical Obstetrics and Gynecology, 57(4), 659–673. 10.1097/grf.0000000000000059 25264697 PMC4216627

[hsc14094-bib-0012] Dunlop, A. L. , Adams, E. K. , Hawley, J. , Blake, S. C. , & Joski, P. (2016). Georgia's Medicaid family planning waiver: Working together with Title X to enhance access to and use of contraceptive and preventive health services. Women's Health Issues, 26(6), 602–611. 10.1016/j.whi.2016.07.006 27599676

[hsc14094-bib-0013] Finer, L. B. , Sonfield, A. , & Jones, R. K. (2014). Changes in out‐of‐pocket payments for contraception by privately insured women during implementation of the federal contraceptive coverage requirement. Contraception, 89, 97–102. 10.1016/j.contraception.2013.11.015 24332745

[hsc14094-bib-0014] Finer, L. B. , & Zolna, M. R. (2011). Unintended pregnancy in the United States: Incidence and disparities, 2006. Contraception, 84(5), 478–485. 10.1016/j.contraception.2011.07.013 22018121 PMC3338192

[hsc14094-bib-0015] Finer, L. B. , & Zolna, M. R. (2016). Declines in unintended pregnancy in the United States, 2008–2011. New England Journal of Medicine, 374(9), 843–852. 10.1056/nejmsa1506575 26962904 PMC4861155

[hsc14094-bib-0016] Fowler, C. I. , Lloyd, S. , Gable, J. , Wang, J. , & McClure, E. (2012). Family planning annual report: 2011 National summary. Research Triangle Park. https://opa.hhs.gov/sites/default/files/2020‐07/fpar‐2011‐national‐summary.pdf

[hsc14094-bib-0017] Frost, J. J. , Gold, R. B. , & Bucek, A. (2012). Specialized family planning clinics in the United States: Why women choose them and their role in meeting women's health care needs. Women's Health Issues, 22(6), e519–e525. 10.1016/j.whi.2012.09.002 23122212

[hsc14094-bib-0018] Frost, J. J. , Sonfield, A. , & Gold, R. B. (2006). Estimating the impact of expanding Medicaid eligibility for family planning services. Guttmacher Institute. https://www.guttmacher.org/sites/default/files/pdfs/pubs/2006/08/16/or28.pdf

[hsc14094-bib-0019] Gibbs, S. , Harvey, S. M. , Bui, L. , Oakley, L. , Luck, J. , & Yoon, J. (2020). Evaluating the effect of Medicaid expansion on access to preventive reproductive care for women in Oregon. Preventive Medicine, 130, 105899. 10.1016/j.ypmed.2019.105899 31730946

[hsc14094-bib-0020] Gipson, J. D. , Koenig, M. A. , & Hindin, M. J. (2008). The effects of unintended pregnancy on infant, child, and parental health: A review of the literature. Studies in Family Planning, 39(1), 18–38. 10.1111/j.1728-4465.2008.00148.x 18540521

[hsc14094-bib-0021] Gomez, A. M. , Fuentes, L. , & Allina, A. (2014). Women or LARC first? Reproductive autonomy and the promotion of long‐acting reversible contraceptive methods. Perspectives on Sexual and Reproductive Health, 46(3), 171–175. 10.1363/46e1614 24861029 PMC4167937

[hsc14094-bib-0022] Guttmacher Institute . (2015). Contraceptive use in the United States . Retrieved January 5, 2021 from https://www.guttmacher.org/sites/default/files/pdfs/pubs/fb_contr_use.pdf

[hsc14094-bib-0023] Guttmacher Institute . (n.d.). 2012–2014 Continuity and Change in Contraceptive Use study . Retrieved February 24, 2021 from https://www.guttmacher.org/population‐center/dataset/2012‐2014‐continuity‐and‐change‐contraceptive‐use‐study#

[hsc14094-bib-0024] Guzzo, K. B. , & Hayford, S. R. (2018). Adolescent reproductive and contraceptive knowledge and attitudes and adult contraceptive behavior. Maternal and Child Health Journal, 22(1), 32–40. 10.1007/s10995-017-2351-7 28755044 PMC5764783

[hsc14094-bib-0025] Hale, N. , Khoury, A. , & Smith, M. (2018). Use of highly effective reversible contraception in Title X clinics: Variation by selected state characteristics. Women's Health Issues, 28(4), 289–296. 10.1016/j.whi.2018.03.003 29661696

[hsc14094-bib-0026] Hamidi, O. P. , Deimling, T. , Lehman, E. , Weisman, C. , & Chuang, C. (2018). High self‐efficacy is associated with prescription contraceptive use. Women's Health Issues, 28(6), 509–513. 10.1016/j.whi.2018.04.006 30131220 PMC6345511

[hsc14094-bib-0027] Holt, K. , Dehlendorf, C. , & Langer, A. (2017). Defining quality in contraceptive counseling to improve measurement of individuals' experiences and enable service delivery improvement. Contraception, 96, 133–137. 10.1016/j.contraception.2017.06.005 28645786

[hsc14094-bib-0028] Holt, K. , Reed, R. , Crear‐Perry, J. , Scott, C. , Wulf, S. , & Dehlendorf, C. (2020). Beyond same‐day long‐acting reversible contraceptive access: A person‐centered framework for advancing high‐quality, equitable contraceptive care. American Journal of Obstetrics & Gynecology, 222(4), S878.e1–S878.e6. 10.1016/j.ajog.2019.11.1279 31809706

[hsc14094-bib-0029] Johnston, E. M. , & McMorrow, S. (2020). The relationship between insurance coverage and use of prescription contraception by race and ethnicity: Lessons from the Affordable Care Act. Women's Health Issues, 30(2), 73–82. 10.1016/j.whi.2019.11.005 31889615

[hsc14094-bib-0030] Johnston, E. M. , Strahan, A. E. , Joski, P. , Dunlop, A. L. , & Adams, K. (2018). Impacts of the affordable care act's Medicaid expansion on women of reproductive age: Differences by parental status and state policies. Women's Health Issues, 28(2), 122–129. 10.1016/j.whi.2017.11.005 29275063

[hsc14094-bib-0031] Jones, R. K. (2017). Change and consistency in U.S. women's pregnancy attitudes and associations with contraceptive use. Contraception, 95(5), 485–490. 10.1016/j.contraception.2017.01.009 28137557 PMC5466839

[hsc14094-bib-0032] Jones, R. K. (2018). Is pregnancy fatalism normal? An attitudinal assessment among women trying to get pregnant and those not using contraception. Contraception, 98, 255–259. 10.1016/j.contraception.2018.05.015 29792840 PMC6139273

[hsc14094-bib-0033] Jones, R. K. , Tapales, A. , Lindberg, L. D. , & Frost, J. (2015). Using longitudinal data to understand changes in consistent contraceptive use. Perspectives on Sexual and Reproductive Health, 47(3), 131–139. 10.1363/47e4615 26287965 PMC4976085

[hsc14094-bib-0034] Kavanaugh, M. L. , Douglas‐Hall, A. , & Finn, S. M. (2020). Health insurance coverage and contraceptive use at the state level: Findings from the 2017 Behavioral Risk Factor Surveillance System. Contraception, 2, 1–7. 10.1016/j.conx.2019.100014 PMC7286150 32550529

[hsc14094-bib-0035] Kavanaugh, M. L. , & Pliskin, E. (2020). Use of contraception among reproductive‐aged women in the United States, 2014 and 2016. F&S Reports, 1(2), 83–93. 10.1016/j.xfre.2020.06.006 34223223 PMC8244260

[hsc14094-bib-0036] Kennedy, C. E. , Yeh, P. T. , Gonsalves, L. , Jafri, H. , Faffield, M. E. , Kiarie, J. , & Narasimhan, M. L. (2019). Should oral contraceptive pills be available without a prescription? A systematic review of over‐the‐counter and pharmacy access availability. BMJ Global Health, 4, 1–17. 10.1136/bmjgh-2019-001402 PMC6606062 31321085

[hsc14094-bib-0037] Langston, A. M. , Rosario, L. , & Westhoff, C. L. (2010). Structured contraceptive counseling—A randomized controlled trial. Patient Education and Counseling, 81, 362–367. 10.1016/j.pec.2010.08.006 20869187

[hsc14094-bib-0038] Leathers, S. J. , & Kelley, M. A. (2000). Unintended pregnancy and depressive symptoms among first‐time mothers and fathers. American Journal of Orthopsychiatry, 70(4), 523–531. 10.1037/h0087671 11086530

[hsc14094-bib-0039] Lee, J. , Papic, M. , Baldauf, E. , Updike, G. , & Schwarz, E. B. (2015). A checklist approach to caring for women seeking pregnancy testing: Effects on contraceptive knowledge and use. Contraception, 91(2), 143–149. 10.1016/j.contraception.2014.11.003 25492313 PMC4303533

[hsc14094-bib-0040] Levesque, J. , Harris, M. F. , & Russell, G. (2013). Patient‐centred access to health care: Conceptualising access at the interface of health systems and populations. International Journal for Equity in Health, 12(18), 1–9. 10.1186/1475-9276-12-18 23496984 PMC3610159

[hsc14094-bib-0041] Li, C. (2014). *The performance of MLR*, *USLMV*, *and WLSMV estimation in structural regression models with ordinal variables* [Doctoral dissertation, Michigan State University]. https://d.lib.msu.edu/etd/3268

[hsc14094-bib-0042] Mutua, M. M. , Achia, T. N. O. , Manderson, L. , & Musenge, E. (2019). Spatial and socio‐economic correlates of effective contraception among women seeking post‐abortion care in healthcare facilities in Kenya. PLoS One, 14(3), 1–28. 10.1371/journal.pone.0214049 PMC6436713 30917161

[hsc14094-bib-0043] Mwaikambo, L. , Speizer, I. S. , Schurmann, A. , Morgan, G. , & Fikree, F. (2011). What works in family planning interventions: A systematic review. Studies in Family Planning, 42(2), 67–82. 10.1111/j.1728-4465.2011.00267.x 21834409 PMC3761067

[hsc14094-bib-0044] National Health Service . (2020). How effective is contraception at preventing pregnancy? Retrieved January 5, 2021 from https://www.nhs.uk/conditions/contraception/how‐effective‐contraception/

[hsc14094-bib-0045] Nearns, J. (2009). Health insurance coverage and prescription contraceptive use among young women at risk for unintended pregnancy. Contraception, 79, 105–110. 10.1016/j.contraception.2008.08.004 19135566

[hsc14094-bib-0046] Nussbeck, F. W. , Eid, M. , & Lischetzke, T. (2006). Analysing multitrait‐multimethod data with structural equation models for ordinal variables applying the WLSMV estimator: What sample size is needed for valid results? British Journal of Mathematical and Statistical Psychology, 59, 195–213. 10.1348/000711005X67490 16709286

[hsc14094-bib-0047] O'Rourke, K. , Richman, A. , Roddy, M. , & Custer, M. (2008). Does pregnancy/paternity intention predict contraception use? A study among US soldiers who have completed initial entry training. Journal of Family Planning and Reproductive Health Care, 34(3), 165–168. 10.1783/147118908784734891 18577315

[hsc14094-bib-0048] Pratt, R. , Stephenson, J. , & Mann, S. (2014). What influences contraceptive behaviour in women who experience unintended pregnancy? A systematic review of qualitative research. Journal of Obstetrics and Gynaecology, 34(8), 693–699. 10.3109/01443615.2014.920783 24911041

[hsc14094-bib-0049] Redhead, C. S. , & Kinzer, J. (2015). Implementing the affordable care act: Delays, extensions, and other actions taken by the administration. Congressional Research Service. https://fas.org/sgp/crs/misc/R43474.pdf

[hsc14094-bib-0050] Reid, A. K. , & Allum, N. (2019). Learn about analysing age in survey data using polynomial regression in R with data from the British Crime Survey (2007). SAGE Research Methods Datasets. 10.4135/9781526489098

[hsc14094-bib-0051] Rocca, C. H. , & Harper, C. C. (2012). Do racial and ethnic differences in contraceptive attitudes and knowledge explain disparities in method use? Perspectives on Sexual and Reproductive Health, 44(3), 150–158. 10.1363/4415012 22958659

[hsc14094-bib-0052] Schwarz, E. B. , Smith, R. , Steinauer, J. , Reeves, M. F. , & Caughey, B. (2008). Measuring the effects of unintended pregnancy on women's quality of life. Contraception, 78(3), 204–210. 10.1016/j.contraception.2008.04.120 18692610 PMC2580059

[hsc14094-bib-0053] Smith, C. , Gold, L. , Ngo, T. D. , Sumpter, C. , & Free, C. (2015). Mobile phone‐based interventions for improving contraceptive use. Cochrane Database of Systematic Reviews, 6, 1–43. 10.1002/14651858.CD011159.pub2 PMC6485989 26115146

[hsc14094-bib-0054] Sonfield, A. (2011). New federal protections expand coverage without cost‐sharing of contraceptives and other women's preventive services. Guttmacher Policy Review, 14(3), 24. https://www.guttmacher.org/gpr/2011/08/new‐federal‐protections‐expand‐coverage‐without‐cost‐sharing‐contraceptives‐and‐other

[hsc14094-bib-0055] Sonfield, A. , & Gold, R. B. (2011). Medicaid family planning expansions: Lessons learned and implications for the future. Guttmacher Institute. www.guttmacher.org/pubs/Medicaid‐Expansions.pdf

[hsc14094-bib-0056] Sonfield, A. , Tapales, A. , Jones, R. K. , & Finer, L. B. (2015). Impact of the federal contraceptive coverage guarantee on out‐of‐pocket payments for contraceptives: 2014 update. Contraception, 91, 44–48. 10.1016/j.contraception.2014.09.006 25288034 PMC4712914

[hsc14094-bib-0057] Steinberg, J. R. , Tschann, J. M. , Henderson, J. T. , Drey, E. A. , Steinauer, J. E. , & Harper, C. C. (2013). Psychological distress and post‐abortion contraceptive method effectiveness level chosen at an urban clinic. Contraception, 88(6), 1–16. 10.1016/j.contraception.2013.08.009 24094755 PMC3844792

[hsc14094-bib-0058] Sundstrom, B. , DeMaria, A. L. , Ferrara, M. , Meier, S. , & Billings, D. (2019). “The closer, the better:” The role of telehealth in increasing contraceptive access among women in rural South Carolina. Maternal and Child Health Journal, 23, 1196–1205. 10.1007/s10995-019-02750-3 31228142

[hsc14094-bib-0059] Swan, L. E. , Auerbach, S. L. , Ely, G. E. , Agbemenu, K. , Mencia, J. , & Araf, N. R. (2020). Family planning practices in Appalachia: Focus group perspectives on service needs in the context of regional substance abuse. International Journal of Environmental Research and Public Health, 17(4), 1198. 10.3390/ijerph17041198 32069932 PMC7068406

[hsc14094-bib-0060] Trussell, J. (2011). Contraceptive failure in the United States. Contraception, 83, 397–404. 10.1016/j.contraception.2011.01.021 21477680 PMC3638209

[hsc14094-bib-0061] Urrutia, R. P. , Polis, C. B. , Jensen, E. T. , Greene, M. E. , Kennedy, E. , & Stanford, J. B. (2018). Effectiveness of fertility awareness‐based methods for pregnancy prevention: A systematic review. Obstetrics & Gynecology, 132(3), 591–604. 10.1097/aog.0000000000002784 30095777

[hsc14094-bib-0062] Wright, D. (2003). National Survey of Families and Households (P9238): Wave 3 field report. University of Wisconcin Survey Center.

[hsc14094-bib-0063] Zimmerman, M. S. (2017). Information poverty and reproductive healthcare: Assessing the reasons for inequity between income groups. Social Work in Public Health, 32(3), 210–221. 10.1080/19371918.2016.1268990 28129076

