# Supplementary material for: Pathways to reproductive autonomy: Using path analysis to predict family planning outcomes in the United States
Source: Health Soc Care Community. 2022 Nov 1;30(6):e6487–99. doi: 10.1111/hsc.14094 (PMC10092462; doi:10.1111/hsc.14094)
Supplement: Supplementary file 1 — Appendix S1–S3 [file HSC-30-e6487-s001.docx]

1. Appendix

**Hypotheses for Path Analysis Model are Shown in** Figure 1.

**Hypothesis** **1:** Lower levels of pregnancy fatalism, having insurance, experiencing provider relational and informative provider engagement, living in a state with expanded family planning Medicaid and having greater knowledge about birth control will predict optimal contraceptive access.

**Hypothesis** **2:** Lower levels of pregnancy fatalism, having insurance, having greater knowledge about birth control, having sex more frequently and having optimal contraceptive access will predict increased contraceptive use.

**Hypothesis** **3:** Having less knowledge about birth control, having sex less frequently, having optimal contraceptive access and increased contraceptive use will predict lower rates of unintended pregnancy.

**Hypothesis** **4:** Pregnancy fatalism, insurance status, relational and informative provider engagement, family planning Medicaid expansion and contraceptive knowledge will have an indirect effect on contraceptive use via contraceptive access.

**Hypothesis** **5:** Pregnancy fatalism, insurance status, relational and informative provider engagement, family planning Medicaid expansion (hypothesis 5 E) and contraceptive knowledge will have an indirect effect on unintended pregnancy via contraceptive access and contraceptive use.

**Hypothesis** **6:** Pregnancy fatalism, insurance status, contraceptive knowledge, frequency of sex and contraceptive access will have an indirect effect on unintended pregnancy via contraceptive use.

**Hypothesis** **7:** Pregnancy fatalism, insurance status, relational and informative provider engagement, family planning Medicaid expansion and contraceptive knowledge will have an indirect effect on unintended pregnancy via contraceptive access.

1. Appendix

Table 1 Attrition from Time One to Time Four (*N* = 4634)

|  | *M* (*SD*) or *n* (%) | | |  | |
| --- | --- | --- | --- | --- | --- |
| Variable at Time One | Stayed (*n* = 1842) | Dropped out (*n* = 2792) | Test Statistic | | Sig. |
| Age | 28.62 (5.31) | 28.13 (5.27) | *t*(4632) = 3.074 | | 0.002** |
| Race/ethnicity |  |  | χ^2^(3) = 72.889 | | <0.001*** |
| Black | 150 (31.4%) | 328 (68.6%) |  | |  |
| Hispanic | 261 (29.7%) | 619 (70.3%) |  | |  |
| White | 1265 (43.5%) | 1643 (56.5%) |  | |  |
| Other | 166 (45.1%) | 202 (54.9%) |  | |  |
| Education |  |  | χ^2^(2) = 100.449 | | <0.001*** |
| High school or less | 293 (30.7%) | 662 (69.3%) |  | |  |
| Some college | 627 (35.6%) | 1133 (64.4%) |  | |  |
| Bachelor's degree or higher | 922 (48.0%) | 997 (52.0%) |  | |  |
| Pregnancy fatalism | 1.68 (1.50) | 1.94 (1.53) | *t*(4599) = −5.615 | | <0.001*** |
| Informational provider engagement (yes) | 758 (38.6%) | 1204 (61.4%) | χ^2^(1) = 1.768 | | 0.184 |
| Relational provider engagement (yes) | 1085 (40.9%) | 1570 (59.1%) | χ^2^(1) = 3.237 | | 0.072 |
| Family planning Medicaid expansion (yes) | 1351 (39.0%) | 2113 (61.0%) | χ^2^(1) = 3.210 | | 0.073 |
| Contraceptive knowledge | 3.20 (1.00) | 3.26 (1.08) | *t*(4106.70) = −1.66 | | 0.098 |
| Insurance status (insured) | 1465 (41.7%) | 2048 (58.3%) | χ^2^(1) = 20.233 | | <0.001*** |
| Access to preferred contraception (yes) | 894 (39.5%) | 1268 (56.1%) | χ^2^(1) = 5.740 | | 0.017* |
| Frequency of sex | 1.20 (1.30) | 2.07 (1.32) | *t*(4585) = −1.745 | | 0.081 |
| Contraceptive use | 1.87 (1.46) | 1.51 (1.51) | *t*(4010.27) = 2.85 | | 0.004** |
| Unintended pregnancy (yes) | 509 (32.4%) | 1062 (67.6%) | χ^2^(1) = 54.178 | | <0.001*** |

**p* < 0.05; ***p* < 0.01; ****p* < 0.001.

Table 2 Describing Study's Missing Data, from all Wave Four Participants (*N* = 1247) to Final Study Sample (*N* = 1036)

| Variable | Missing Data | | | | | | |
| --- | --- | --- | --- | --- | --- | --- | --- |
|  | T1 Pregnancy fatalism (PF) | T1 Contraceptive knowledge (CK) | T1 Insurance status (IS) | T2 Access to Preferred Contraceptive Method (Access) | T3 Frequency of sex | T3 Contraceptive use (CU) | T4 Unintended pregnancy (UP) |
| Missing: *n* (%) | 6 (0.5%) | 6 (0.5%) | 9 (0.7%) | 183 (14.7%) | 13 (1.0%) | 3 (0.2%) | 2 (0.2%) |
| Age | *t*(5.4) = 6.28** | *t*(1245) = 1.13 | *t*(1245) = −0.39 | *t*(238.03) = −3.80*** | *t*(1245) = −0.90 | *t*(1245) = 1.33 | *t*(1244) = 33.46*** |
| Race/ethnicity | χ^2^(3) = 18.97*** | χ^2^(3) = 14.74** | χ^2^(3) = 9.13* | χ^2^(3) = 9.67* | χ^2^(3) = 11.98** | χ^2^(3) = 15.84** | χ^2^(3) = 3.81 |
| Education | χ^2^(2) = 0.03 | χ^2^(2) = 1.79 | χ^2^(2) = 3.78 | χ^2^(2) = 5.56 | χ^2^(2) = 2.78 | χ^2^(2) = 3.03 | χ^2^(2) = 2.83 |
| T1 PF | -- | *t*(1239) = −1.20 | *t*(1239) = −0.86 | *t*(1239) = −0.86 | *t*(1239) = −0.45 | *t*(1239) = −1.60 | *t*(1239) = −2.10* |
| T1 CK | *t*(1239) = −0.09 | -- | *t*(1239) = −0.49 | *t*(1239) = 1.44 | *t*(1239) = 1.96 | *t*(1239) = 0.98 | *t*(1239) = 1.05 |
| T1 IS | χ^2^(1) = 0.99 | χ^2^(1) = 1.24 | -- | χ^2^(1) = 17.87*** | χ^2^(1) = 1.21 | χ^2^(1) = 1.15 | χ^2^(1) = 1.15 |
| T1 Access | χ^2^(1) = 0.27 | χ^2^(1) = 0.01 | χ^2^(1) = 0.08 | χ^2^(1) = 5.17* | χ^2^(1) = 1.95 | χ^2^(1) = 0.93 | χ^2^(1) = 2.16 |
| T2 Access | -- | -- | -- | -- | χ^2^(1) = 0.03 | χ^2^(1) = 1.10 | χ^2^(1) = 1.83 |
| T3 Access | -- | -- | -- | -- | χ^2^(1) = 0.05 | † | χ^2^(1) = 0.04 |
| T1 CU | *t*(1238) = 0.57 | *t*(1238) = 1.87 | *t*(1238) = −0.28 | *t*(1238) = 17.53*** | *t*(1238) = −0.64 | *t*(1238) = 0.15 | *t*(1238) = 0.52 |
| T2 CU | -- | -- | -- | *t*(687.2) = 44.70*** | *t*(12.4) = −2.04 | *t*(1242) = 1.31 | *t*(1242) = 0.57 |
| T3 CU | -- | -- | -- | -- | *t*(1242) = −0.67 | -- | *t*(1242) = 0.62 |
| T1 UP | χ^2^(1) = 0.41 | χ^2^(1) = 1.37 | χ^2^(1) = 0.11 | χ^2^(1) = 0.66 | χ^2^(1) = 1.11 | χ^2^(1) = 0.04 | χ^2^(1) = 0.46 |
| T2 UP | -- | -- | -- | χ^2^(1) = 0.00 | χ^2^(1) = 0.45 | χ^2^(1) = 0.10 | χ^2^(1) = 0.07 |
| T3 UP | -- | -- | -- | -- | χ^2^(1) = 0.48 | χ^2^(1) = 0.12 | χ^2^(1) = 0.08 |

**p* < 0.05; ***p* < 0.01; ****p* < 0.001.

†No test statistics computed because the measure of association is a constant.

T1 = Time 1; T2 = Time 2; T3 = Time 3; T4 = Time 4; PF=Pregnancy fatalism; CK=Contraceptive knowledge; IS=Insurance status; Access = Ability to access to preferred contraceptive method; CU = effectiveness of contraceptive method(s) in use; UP=Unintended pregnancy.

1. Appendix

***Path Analysis Direct Effect Estimates.***

*Note:* Solid lines denote significant direct effects; dotted lines denote non-significant direct effects. Model adjusted for the effects of age, age^2^, race/ethnicity and education.
T1 = Time 1; T2 = Time 2; T3 = Time 3; T4 = Time 4
